# Case Report: A novel heterozygous nonsense mutation in *KRIT1* cause hereditary cerebral cavernous malformation

**DOI:** 10.3389/fonc.2023.1141488

**Published:** 2023-04-27

**Authors:** Zhenxing Liu, Kaikai Guo, Xuebin Hu, Xianqin Zhang

**Affiliations:** ^1^ Key Laboratory of Molecular Biophysics of the Ministry of Education, College of Life Science and Technology and Center for Human Genome Research, Huazhong University of Science and Technology, Wuhan, Hubei, China; ^2^ Department of Neurosurgery, Union Hospital, Tongji Medical College, Huazhong University of Science and Technology, Wuhan, China

**Keywords:** cerebral cavernous malformation, vascular malformation disease, *KRIT1*, whole exome sequencing, novel mutation

## Abstract

Cerebral cavernous malformation (CCM) is a vascular malformation of the central nervous system and mainly characterized by enlarged capillary cavities without intervening brain parenchyma. Genetic studies have identified three disease-causing genes (*CCM1/KRIT1*, *CCM2/MGC4607* and *CCM3/PDCD10*) responsible for CCM. Here, we characterized a four-generation family diagnosed with CCM and identified a novel heterozygous mutation c.1159C>T, p.Q387X in *KRIT1* gene by whole exome sequencing and Sanger sequencing. The Q387X mutation resulted in premature termination of KRIT1 protein, which was predicted to be deleterious by the ACMG/AMP 2015 guideline. Our results provide novel genetic evidence support that *KRIT1* mutations cause CCM, and are helpful to the treatment and genetic diagnosis of CCM.

## 1 Introduction

Cerebral cavernous malformation (CCM, OMIM #116860) is a cerebrovascular disease mainly characterized by epileptic seizures, cerebral hemorrhage, and focal neurological deficits (1, 2). In addition, CCM patients also have extraneurological manifestations, including retina, skin, liver, and kidney involvement (1, 2). Epidemiological surveys showed that the prevalence of CCM is about 0.5%, but it is asymptomatic in up to 50%-70% of cases (1, 2). CCM is divided into sporadic (80%) and familial (20%) forms (2). At present, it has been reported that the pathogenic genes are CCM1/*KRIT1*, CCM2/*MGC4607* and CCM3/*PDCD10*, which have autosomal dominant inheritance (2). *KRIT1* mutations account for 53-65% of CCM familial cases (3).

*KRIT1* gene encodes Krev interaction trapped protein 1, which consists of NUDIX domain, multiple NPxY/F (Asn-Pro-X-Tyr/Phe) motifs, four ankyrin repeats (ANK), and a band 4.1/ezrin/radixin/moesin (FERM) domain, and play critical roles in angiogenesis, cell proliferation, maintain the integrity of endothelial junctions, and cell polarity (2). In 1999, Couteulx Sl et al. reported that *KRIT1* mutation led to CCM1, but with incomplete penetrance, and the patient’s phenotype had strong heterogeneity (4, 5). KRIT1 participates in the regulation of RhoA and Cdc42 pathways through β-catenin, HEG1, Rap1, and Rasip1, thereby regulating cell adhesion and migration, playing an important role in vascular development (2). Gault J et al. identified somatic deletion and germline mutation in *KRIT1* gene in CCM1 patients, strongly supporting the “Knudson double hit mechanism” (6). It has been reported that *KRIT1* mutations are mostly null mutations, and KRIT1 mRNA decay has been found in CCM1 patients, so haploinsufficiency may be a potential mechanism of CCM1 (7). Cheng D et al. performed brain magnetic resonance imaging (MRI) on fetuses carrying *KRIT1* mutations, showing that brain MRI has prominent sensitivity and specificity for detecting fetal CCM (8).

Notably, *krit1* heterozygous deletion mice did not have any vascular lesions, and homozygous mutant embryos had vascular defects in early pregnancy and died in the second trimester (9, 10). Deletion of *krit1* in zebrafish embryos resulted in thinning of vessel walls, which was analogous to human and mouse phenotypes (11). Here, we recruited a family of CCM to identify the disease-causing gene.

## 2 Case presentation

**Figure 1:**
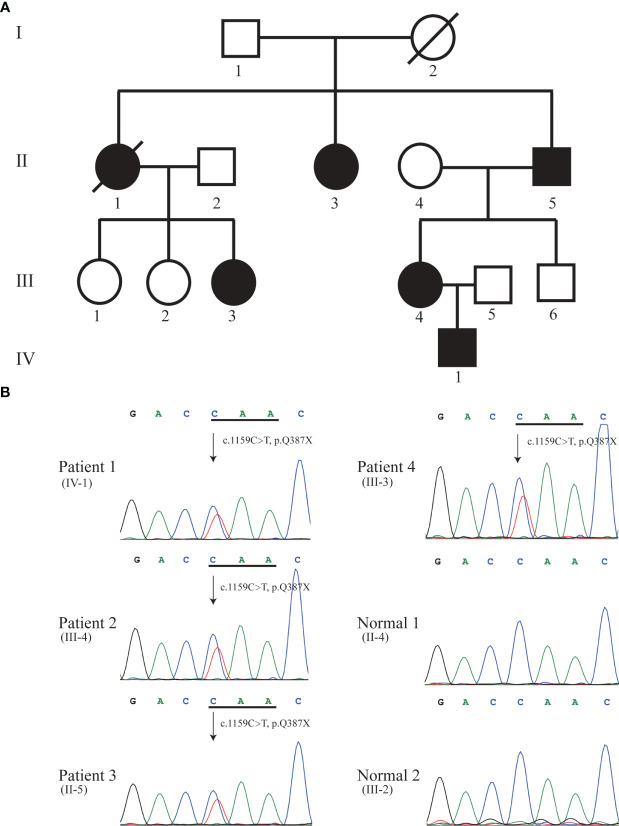
Pedigree **(A)** and Sanger sequencing **(B)**. Sanger DNA sequencing confirmed the heterozygous nonsense mutation c.1159C>T (p.Q387X) in *KRIT1* gene in patient 1 (IV-1), 2 (III-4), 3 (II-5), and 4 (III-3). Solid black symbol indicates patient; a slash indicates deceased individual.

**Figure 2:**
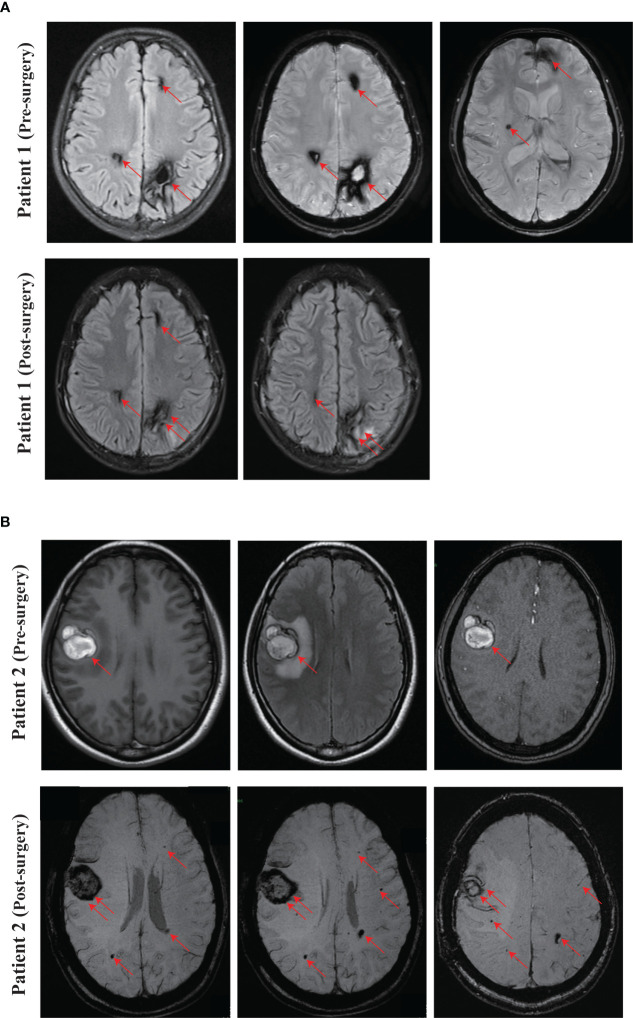
Representing images of CCM. **(A)** SWI images of the proband (Patient 1, IV-1) at pre-surgery and 3 months after surgery. **(B)** Pre-surgery cerebral MRI images of the proband’s mother (Patient 2, III-4), and SWI images at one month after surgery. Red arrow, CCM lesion. Double red arrows, surgical site.

We recruited a family with CCM, at least six individuals were diagnosed with CCM in the family. The proband (Patient 1, IV-1) is a 13-year-old boy, developed intermittent head shaking towards the right side at the beginning of 2021 (**Figure 1A**). The episodes lasted for 1-2 seconds and were not accompanied by loss of consciousness or limb jerking. He was diagnosed with focal seizures at a local hospital and later confirmed to have cavernous malformation through susceptibility weighted imaging (SWI) (**Figure 2A**). In October 2022, the patient’s symptoms worsened, and he experienced generalized seizures. During seizures, his limbs jerked and he had impaired consciousness and was unresponsive. The patient experienced 2-3 such seizures per week, each lasting 1-2 minutes, but he could recover spontaneously. After two weeks of preoperative preparation, the patient underwent surgical treatment with transcranial base resection of cavernous malformation. During the surgery, we found a mulberry-like lesion measuring 2.5 cm×2.5 cm×3 cm in the left parietal lobe. Pathological examination revealed a cavernous malformation with calcification and deposition of yellow iron-containing pigments in the surrounding brain tissue. After surgery, the patient recovered well and did not experience any seizures or other symptoms. The patient continued to take sodium valproate and levetiracetam tablets after discharge. During the 3-month follow-up period, the patient stopped taking medication and did not experience any seizures, and the brain SWI showed good postoperative recovery (**Figure 2A**).

The mother of the proband (Patient 2, III-4, 38 years old) presented with focal seizures in February 2015, cerebral MRI imaging showed a cerebral cavernous angioma in the right temporal lobe (**Figures 1A**, **2B**), and then underwent surgical treatment at the hospital. The SWI images of patient 2 revealed multiple cavernous angioma at one month after surgery (**Figure 2B**). The proband’s grandfather (Patient 3, II-5, 63 years old) was diagnosed with multiple microbleeds foci by SWI (**Figure 1A**). In addition, patient 4 (III-3, 35 years old) were diagnosed with CCM several years ago and underwent surgery in hospital, but no other information is available (**Figure 1A**). Patient 5 (II-1, 57 years old) was diagnosed with multiple CCM before death and presented with hemiparesis, with imaging studies showing up to hundreds of cavernous malformations in the brain (**Figure 1A**). Patient 6 (II-3, 66 years old) was diagnosed with CCM in another hospital, but unfortunately no imaging data was available (**Figure 1A**).

We performed whole-exome sequencing (WES) in the proband. By analyzing the data of whole-exome sequencing, we identified a novel heterozygous nonsense mutation (NM_194454.3: c.1159C>T, p.Q387X) in exon 12 of the *KRIT1* gene in the proband (Patient 1) (**Figure 1B**). We further to confirm whether the identified mutation segregates in the family by Sanger DNA sequencing. This result showed that the proband’s mother (Patient 2, III-4), grandfather (Patient 3, II-5), and aunt (Patient 4, III-3) carries the mutation (**Figure 1B**). No mutation in *MGC4607* or *PDCD10* was identified in the proband, III-3, III-4, and II-5 in the family. Regrettably, we did not get the blood sample of patients 5 (II-1) and 6 (II-3) (**Figure 1A**). The allele frequency of the c.1159C>T mutation was not found in databases (NHLBI GO Exome Sequencing Project, ExAC and 1000 Genomes). *KRIT1* Q387X mutation leads to produce a premature stop codon, which might make truncated KRIT1 protein or lead to the degradation of *KRIT1* mRNA, and the ACMG/AMP 2015 guideline prediction results showed that the mutation is a pathogenic mutation.

## 3 Discussion

In this study, we identified a novel heterozygous nonsense mutation c.1159C>T (p.Q387X) in *KRIT1* gene in a CCM family. The FERM domain of KRIT1 protein is responsible for binding RAP1 protein, the master regulator of endothelial barrier function and angiogenesis, and HEG1, the regulator of proper localization of KRIT1 protein at endothelial cell–cell junctions (2, 12). The Q387X mutation deletes the FERM domain of the KRIT1 protein, will result in impaired binding of the mutant protein to RAP1 and HEG1 proteins. Furthermore, other null mutations of *KRIT1* have been reported (13, 14), Q387X mutation may also lead to the degradation of *KRIT1* mRNA.

As same as most reported mutations of the *KRIT1* gene, our reference transcript ID is ENST00000394505.7 (NM_194454.3). Mao CY et al. found a nonsense mutation c.1159G>T (p.E387X) in the KRIT1 gene of CCM patients from a Chinese family, but the reference transcript ID is ENST00000394503.6 (12). According to *KRIT1* gene transcript ID: ENST00000394505.7 (NM_194454.3), the mutation site found by Mao CY et al. corresponds to c.1303G>T (p.E435X) (12). Therefore, the c.1159C>T (p.Q387X) mutation in the *KRIT1* gene found in this study is a novel mutation.

Denier C et al. investigated 202 *KRIT1* mutation carriers and found that 37.6% of individuals were symptom-free, T2-weighted MRI examination of 53 asymptomatic *KRIT1* mutation carriers revealed that 43 had CCM lesions (81.1%) (15). It is noteworthy that two asymptomatic *KRIT1* mutation carriers with T2-weighted MRI examination did not show any CCM lesions, but CCM lesions were found by gradient Echo MRI (15). Six patients in the families we investigated carried the *KRIT1* c.1159C>T (p.Q387X) heterozygous mutation.

## 4 Conclusion

In conclusion, we identified a novel heterozygous mutation of *KRIT1* c.1159C>T (p.Q387X), which helps to expand the mutation spectrum of *KRIT1* gene and emphasizes the importance of genetic testing in CCM patients and their families. The combination of genetic testing and MRI can identify subjects at risk of developing CCM, so that take appropriate treatment measures as early as possible, improve the success rate of treatment and reduce the burden of patients.

## Data availability statement

The original contributions presented in the study are included in the article/supplementary material. Further inquiries can be directed to the corresponding authors.

## Ethics statement

The studies involving human participants were reviewed and approved by the ethics committee of Huazhong University of Science and Technology. Written informed consent to participate in this study was provided by the participants’ legal guardian/next of kin. Written informed consent was obtained from the individual(s), and minor(s)’ legal guardian/next of kin, for the publication of any potentially identifiable images or data included in this article.

## Author contributions

All authors contributed to the study conception and design. XH and KG collected clinical data and blood samples. Genetic analysis was conducted by ZL. The first draft of the manuscript was written by ZL and KG. XZ and XH revised the manuscript. All authors read and approved the final manuscript.

## References

[1] Perrelli A Retta SF. Polymorphisms in genes related to oxidative stress and inflammation: emerging links with the pathogenesis and severity of cerebral cavernous malformation disease. Free Radic Biol Med (2021) 172:403–17. doi: 10.1016/j.freeradbiomed.2021.06.021 34175437

[2] Riolo G Ricci C Battistini S. Molecular genetic features of cerebral cavernous malformations (CCM) patients: an overall view from genes to endothelial cells. Cells (2021) 10(3):704. doi: 10.3390/cells10030704 33810005 PMC8005105

[3] Ricci C Cerase A Riolo G Manasse G Battistini S. KRIT1 gene in patients with cerebral cavernous malformations: clinical features and molecular characterization of novel variants. J Mol Neurosci (2021) 71(9):1876–83. doi: 10.1007/s12031-021-01814-w PMC8421287 33651268

[4] Laberge-le Couteulx S Jung HH Labauge P Houtteville JP Lescoat C Cecillon M. Truncating mutations in CCM1, encoding KRIT1, cause hereditary cavernous angiomas. Nat Genet (1999) 23(2):189–93. doi: 10.1038/13815 10508515

[5] Cau M Loi M Melis M Congiu R Loi A Meloni C. C329X in KRIT1 is a founder mutation among CCM patients in Sardinia. Eur J Med Genet (2009) 52(5):344–8. doi: 10.1016/j.ejmg.2009.05.002 19454328

[6] Gault J Shenkar R Recksiek P Awad IA. Biallelic somatic and germ line CCM1 truncating mutations in a cerebral cavernous malformation lesion. Stroke (2005) 36(4):872–4. doi: 10.1161/01.STR.0000157586.20479.fd 15718512

[7] Cavé-Riant F Denier C Labauge P Cécillon M Maciazek J Joutel A. Spectrum and expression analysis of KRIT1 mutations in 121 consecutive and unrelated patients with cerebral cavernous malformations. Eur J Hum Genet (2002) 10(11):733–40. doi: 10.1038/sj.ejhg.5200870 12404106

[8] Cheng D Shang X Gao W Barkhof F Liu Y. Fetal familial cerebral cavernous malformation with a novel heterozygous KRIT1 variation. Neurology (2021) 97(21):986–8. doi: 10.1212/WNL.0000000000012852 34556564

[9] Whitehead KJ Plummer NW Adams JA Marchuk DA Li DY. Ccm1 is required for arterial morphogenesis: implications for the etiology of human cavernous malformations. Development (2004) 131(6):1437–48. doi: 10.1242/dev.01036 14993192

[10] Plummer NW Gallione CJ Srinivasan S Zawistowski JS Louis DN Marchuk DA. Loss of p53 sensitizes mice with a mutation in Ccm1 (KRIT1) to development of cerebral vascular malformations. Am J Pathol (2004) 165(5):1509–18. doi: 10.1016/S0002-9440(10)63409-8 PMC1618670 15509522

[11] Hogan BM Bussmann J Wolburg H Schulte-Merker S. ccm1 cell autonomously regulates endothelial cellular morphogenesis and vascular tubulogenesis in zebrafish. Hum Mol Genet (2008) 17(16):2424–32. doi: 10.1093/hmg/ddn142 18469344

[12] Mao CY Yang J Zhang SY Luo HY Song B Liu YT. Exome capture sequencing identifies a novel CCM1 mutation in a Chinese family with multiple cerebral cavernous malformations. Int J Neurosci (2016) 126(12):1071–6. doi: 10.3109/00207454.2015.1118628 26643368

[13] Zhang F Xue Y Zhang F Wei X Zhou Z Ma Z. Identification of a novel CCM1 frameshift mutation in a Chinese han family with multiple cerebral cavernous malformations. Front Neurosci (2020) 14:525986. doi: 10.3389/fnins.2020.525986 33071727 PMC7538688

[14] Wang H Pan Y Zhang Z Li X Xu Z Suo Y. A novel KRIT1/CCM1 gene insertion mutation associated with cerebral cavernous malformations in a Chinese family. J Mol Neurosci (2017) 61(2):221–6. doi: 10.1007/s12031-017-0881-5 28160210

[15] Denier C Labauge P Brunereau L Cavé-Riant F Marchelli F Arnoult M. Clinical features of cerebral cavernous malformations patients with KRIT1 mutations. Ann Neurol (2004) 55(2):213–20. doi: 10.1002/ana.10804 14755725

